# Experimental Study on Compression Failure of Composite Laminates with Prefabricated Surface Cracks

**DOI:** 10.3390/ma14133616

**Published:** 2021-06-28

**Authors:** Wei Sun, Tian Ouyang, Zengshan Li, Yan Li

**Affiliations:** 1 Chinese Aeronautical Establishment, Beijing 100012, China; liyan@cae.ac.cn; 2 School of Aeronautic Science and Engineering, Beihang University, Beijing 100191, China; zengshanli@buaa.edu.cn

**Keywords:** damage tolerance, composite laminates, surface cracks, crack propagation, compression failure

## Abstract

A new compression test fixture was designed in the present work to study the damage tolerance of composite laminates with surface cracks or notches. The compression failure behaviors of CCF300/5228A quasi-isotropic composite laminates with prefabricated surface cracks were studied experimentally. Through the size design of the test fixture and specimens and an application of a simple test method, the complex crack growth process was captured. The experimental results showed that the compression failure modes were mainly affected by crack angles and depths, and there were two typical failure modes, which were local intra- and inter-laminar damage propagating from the crack tips and delamination growth induced from the crack leading edge. This study verified the validity of the test fixture and test method, and revealed the compression failure mechanisms of composite laminates with surface cracks.

## 1. Introduction

With the wide application of composite materials in wind turbine blades, aircraft structures, and large civil structures, the damage tolerance and durability of composite materials have become key issues to be considered in structural design [1,2,3]. On the one hand, the compressive strength of composites (including fiber tows and unidirectional laminates) is significantly lower than the tensile strength, and the structural buckling induced by the compression load will lead to premature failure of the structure. On the other hand, different from tensile failure of composite laminates with notches and cracks, the formation and extrusion of fracture surface and the local failure of the material due to contact stress in the compression process are more complex. Therefore, it is of important engineering significance to study the damage propagation and failure behaviors of composites with notches and cracks under compressive loading.

There have been many experimental studies on the compression failure of composite materials, among which compression after impact (CAI) based on ASTM D7137 [4] standard is a typical test. The compressive strength of laminates with impact damage can decrease significantly, and the CAI failure is mainly caused by sublaminate buckling and the propagation of severe local damage (fiber breakage or even the penetrated hole) [5,6,7,8,9]. Compared with the impact damage, the failure of laminates with circular notches is mainly affected by stress concentration and many scholars have conducted research on the damage initiation and propagation of notched laminates [10,11,12,13,14]. When it comes to a slender notch, the stress concentration becomes higher, making intra- and inter-laminar damage initiate relatively earlier from the notch tip [15,16,17]. In engineering, a large slender notch that serves a single skin bay and a central stiffener is often used to conservatively address the wide range of possible large damage scenarios [18,19]. Compared with slender notches, real cracks have much higher stress concentration effects due to the sharp crack tips. Matrix cracks [20], delamination cracks, and fiber failure cracks are three common crack types in laminates, and cracks related to matrix properties generally propagate between fibers because it requires a high energy release rate to cut off the fibers and propagate. For fiber failure crack, some scholars used the local high stress at the intra-laminar crack tips in unidirectional laminates to study the compression failure behaviors of fibers [21,22]. For a multi-crack problem, Xu et al. [23] performed a detailed numerical study to understand the damage development at the crack tips of quasi-isotropic laminates. They found the complete failure of laminates did not occur at the time fiber breakage initiated but occurred after a period of progressive fiber breakage in 0° fibers within the damage zone.

Surface crack is a kind of local damage and can be considered notch-like damage that does not penetrate the laminate through the thickness. Its concept comes from the scratch on the structure surface in engineering and most of them are generally 1 or 2 plies deep. For slight scratches, researchers mainly focus on the formation mechanisms and characterization parameters of them [24,25,26]. Under axial tensile load, even 2-ply deep scratches can significantly affect the crack initiation response occurring early in the loading regime of the specimen. A deep scratch will have a significant influence on the strength and failure behaviors of composites by causing a discontinuity in the load bearing plies [27]. The scratch tip (the crack front or the leading edge of the crack in the present study) location within the stacking sequence is a dominant factor for tension load capacity, and it can result in undesirable bending and torsion deformations, further triggering the delamination [28]. Due to the crack passing through the width direction of the test piece in their research, the crack tip propagation in plane was ignored. For composite laminates, the study of dynamic inter- and intra- crack propagation is mainly based on numerical simulation [29,30,31]. There is a correlation between the damage of composite laminates and the intra-crack propagation, especially when in-plane shear nonlinearity and plastic damage are considered. Russell [32] proposed a fracture mechanics-based residual strength model for composite laminates with surface or partial thickness damage under tension (or compression). Due to the complex damage forms not being considered, the application scope of this method is limited. Arikan [33] investigated the failure behavior of composite pipes with surface cracks and the dependence of the burst strengths on the crack angle. In theory, his study corresponds to the failure of composite laminates with surface crack under biaxial tensile loading, and the results showed that crack growth started with delamination.

**Figure 1:**
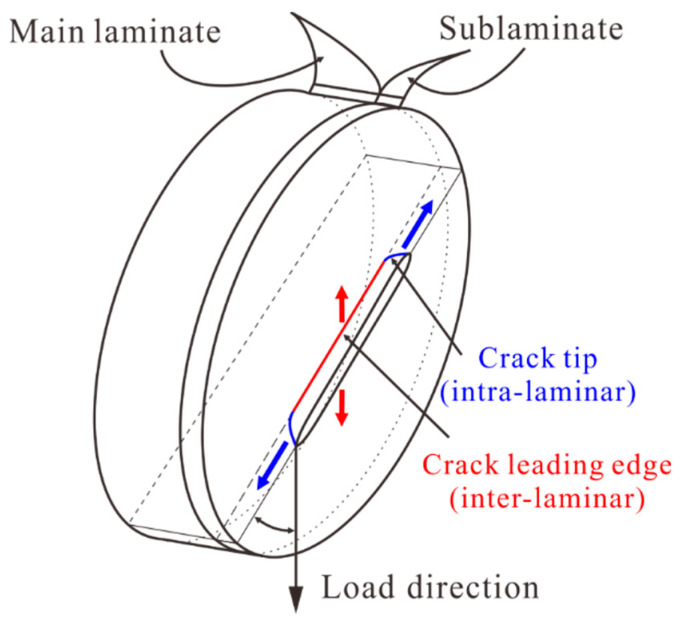
The schematic diagram of the surface crack.

At present, research on laminates with surface cracks mainly focuses on crack propagation (delamination crack) under the tensile load. Little research focuses on the surface crack with sharp crack front and crack tip propagation under the compressive load, as shown in Figure 1. The surface crack has a crack leading edge along the laminate thickness direction, and the crack leading edge may propagate preferentially between plies; meanwhile, due to the constraint of the main laminate on the crack tip of the sublaminate, the crack propagation and the extrusion of crack surface have complex mechanical behaviors. However, there is a lack of testing methods for the compression of laminates with surface cracks, especially at the material level. In this paper, a new compression test fixture was designed based on the test fixtures used in ASTM D7137 and ASTM D695 [34] standards. An efficient test method was proposed to conduct a study on the compression failure of CCF300/5228A quasi-isotropic laminates with prefabricated surface cracks. The validity of the test method was verified, while the crack growth and damage propagation of laminates with different cracks were studied.

## 2. Compression Fixture

**Figure 2:**
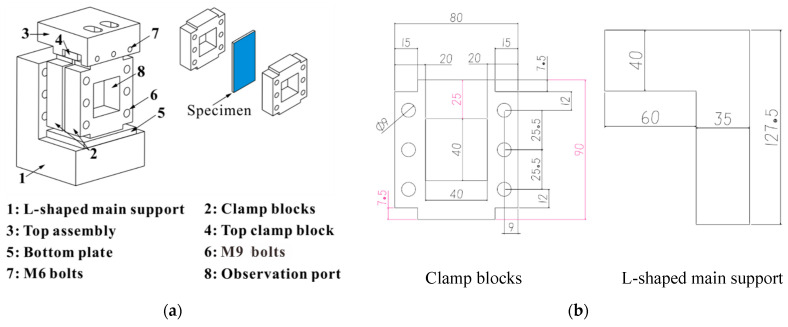
(**a**) The schematic diagram of the compression fixture and (**b**) main dimension parameters of the fixture.

Figure 2a shows the designed test fixture based on the loading and constraint conditions of ASTM D7137 and ASTM D695. The fixture mainly consisted of five parts: (1) an L-shaped main support, (2) two clamp blocks, (3) a top assembly, (4) a top clamp block, and (5) a bottom plate. Two clamp blocks were connected with the L-shaped main support through six M9 bolts and the specimen was clamped by the clamp blocks with a tightening torque of 0.25 N·m (finger tightening). Three M6 bolts were used to clamp the loading end of the specimen through a top clamp block. A bottom plate was placed under the specimen to protect the L-shaped main support from being crushed. The boundary condition of the specimen was similar to the fixed boundary condition, which can restrain the global buckling of the laminate (pre-numerical analysis showed that the fixture could keep a 2 mm thick quasi-isotropic laminate from buckling under 12,000 με). Moreover, in order to observe the damage propagation during compression, there was a 40 mm × 40 mm opening in the clamp plate. Figure 2b shows the main dimension parameters of the fixture.

The acceptance criterion of specimen failure mode was that failure occurred in the test section or the complete propagation of prefabricated crack occurred before the end failure of the specimen.

## 3. Experiment

### 3.1. Specimens

**Figure 3:**
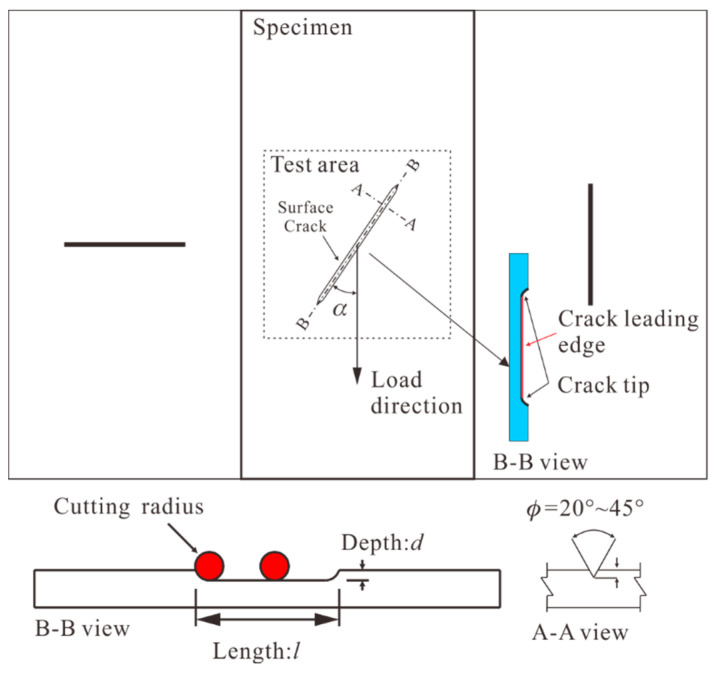
The schematic diagram of the prefabricated crack.

The specimens were 100 mm × 50 mm × 4 mm quasi-isotropic laminates. They were made of CCF300/5228A carbon fiber/epoxy resin unidirectional prepreg with a thickness of 0.125 mm. The elastic parameters of the material were *E*_11_ = 138 GPa, *E*_22_ = 9.04 GPa, *ν*_12_ = 0.307, and *G*_12_ = 3.476 GPa [35]. Figure 3 shows the schematic diagram of the prefabricated crack. A circular saw with a cone angle of about 30° was used to prefabricate the surface cracks. There are three geometric parameters of each crack, which are crack length *l*, depth *d*, and angle *α*.

In the present work, the minimum and maximum crack length were close to 2*h* (*h*, the specimen thickness) and *w*/2 (*w*, the specimen width), respectively, in order to prefabricate enough damage and capture the crack tip propagation. The depth of the crack was about 0.2*h–*0.6*h*. A relatively high crack depth was used, which can prevent the failure of the specimen end. Three typical values of crack angles (0°, 45°, and 90°) were taken from the range of 0°–90°. The test matrix is shown in Table 1. Specimens were divided into nine groups according to different crack lengths and depths, and each group contained three specimens with crack angles of 0°, 45°, and 90°, respectively labeled as #1, #2, and #3.

**Figure 4:**
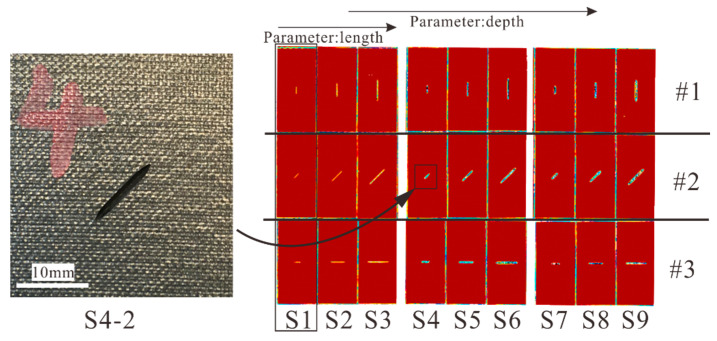
C-scan results of all specimens after crack prefabrication.

After the crack prefabrication, non-destructive inspection was performed to evaluate whether there was unexpected damage induced into the laminates. Figure 4 shows the C-scan results of all specimens. It can be seen that for each specimen, there was no damage other than the prefabricated crack.

### 3.2. Experiment Method

#### 3.2.1. Experiment Equipment

**Figure 5:**
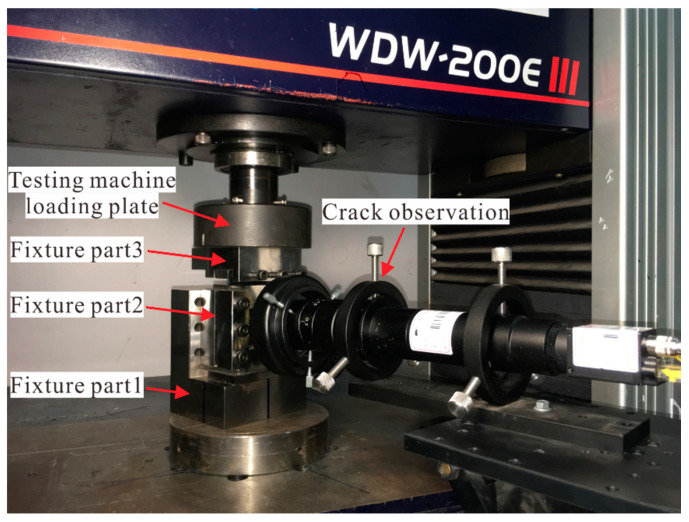
The setup for the compression test of laminates.

As shown in Figure 5, the compression test was carried out on an electronic static testing machine (WDW-200E) (Time Shijin Testing Machine Corp., Ltd., Jinan, China) with a maximum load of 200 kN. The axial load was applied by displacement control with a rate *v* of 1 mm/min. To reduce the influence of test system nonlinearity on the load-displacement curve, each specimen was preloaded to 10 kN to eliminate the internal clearance, and then unloaded to 2 kN (*F*_0_) as the initial test state.

Crack observation equipment was used to capture the crack propagation of the specimens in the compression process, and the sampling interval Δ*t* was set to 0.5 s. By starting the loading and observation equipment at the same time, the corresponding relationship between the loading displacement and the recorded frame could be obtained. After the test, the strain corresponding to different recorded frames was calculated by an equivalent compression strain method.

#### 3.2.2. Equivalent Compression Strain Method

Since the specimen was loaded by a constant displacement rate, the strain increment Δ*ε* between two adjacent frames was constant. Under the linear elasticity assumption, the stress increment could also be considered constant. However, taking into account the influence of material nonlinearity and damage on the compression stiffness of the laminate, the stress increment between the two frames would change. Therefore, Δ*ε* was a better choice to evaluate the equivalent strain level corresponding to the crack growth. The expression of the equivalent compression strain is written as:

(1) $$\varepsilon_{i}=\varepsilon_{0}+\left(i-1\right)\Delta \varepsilon$$

where *i* is the frame number, *ε_i_* is the equivalent compression strain corresponding to *i*, and *ε*_0_ is the compression strain at the initial test state. According to the classical laminate theory, the strain *ε* of a symmetric laminate under the axial load can be calculated as (ignoring the bending effect that may be introduced during compression):

(2) $$\varepsilon =A^{-1}Ν$$

where ***A*** is the in-plane stiffness matrix of the laminate and ***N*** is the in-plane loading matrix.

### 3.3. Determination of ε_0_ and Δε

**Figure 6:**
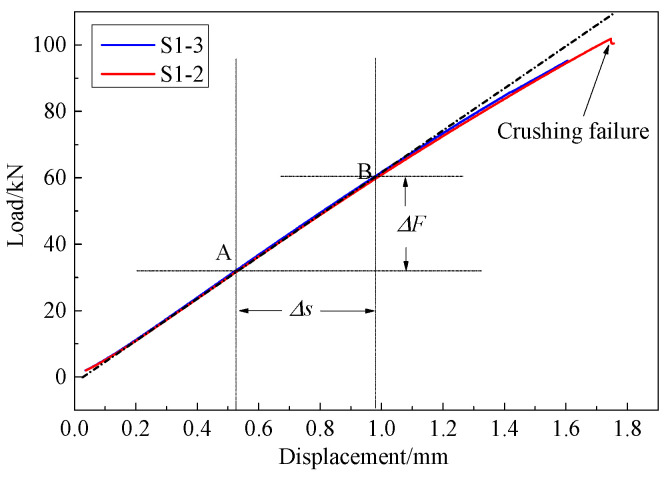
Compression load-displacement curves of specimens with shallow cracks.

Figure 6 shows the load-displacement curves of S1-2 and S1-3. The curves increased linearly up to about 70 kN and then nonlinearity appeared at high loads. Since the curves were quite consistent, the load-displacement curve of S1-2 was taken as an example to illustrate the calculation of the equivalent compressive strain.

The load increment *k* for each frame could be calculated as k=ΔF/ΔN=0.512kN, where ΔN=Δs/(vΔt). Then, based on the classical laminate theory, the strain increment between two adjacent frames could be obtained as Δε=49.6με. Further, *ε*_0_ was also obtained as $\varepsilon_{0}=F_{0}\Delta \varepsilon /k=194\mu \varepsilon$. Finally, the equivalent compression strain was expressed as:

(3) $$\varepsilon_{i}=194+49.6\left(i-1\right)\mu \varepsilon$$

For S1-2, complete crack propagation occurred at the 207th frame, and thus the corresponding strain was 10,412 με, indicating that the failure strain of the specimen with a shallow 45° crack was quite high.

## 4. Results and Discussion

### 4.1. Compression Failure of Specimens with 0° Cracks

**Figure 7:**
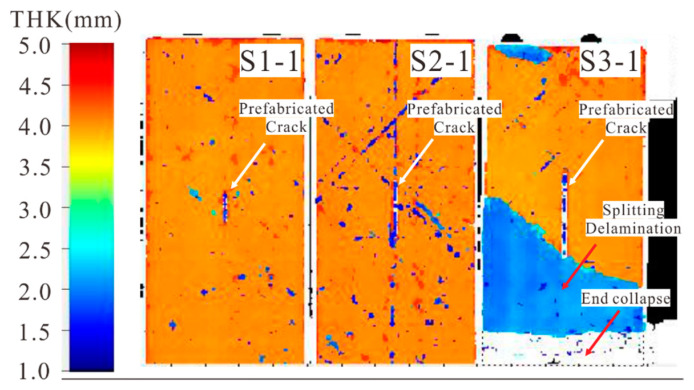
C-scan results of specimens with 0° shallow surface cracks. (THK represents the thickness from which the scanning ultrasonic wave gets reflected.)

Figure 7 shows the C-scan results of specimens with 0° shallow surface cracks. For S3-1, a large area of split delamination (the blue region) and end crushing (the white region) were observed, while no damage propagation occurred near the prefabricated surface crack, which indicates that it is difficult for a 0° shallow surface crack to propagate during compression. The failure of the specimen occurred at the 235th frame, corresponding to a compression strain of about 11,800 με, which is close to the failure strain of the material. Considering the end crushing of S3-1, no compression tests were carried out on S1-1 and S2-1 with shorter prefabricated cracks.

**Figure 8:**
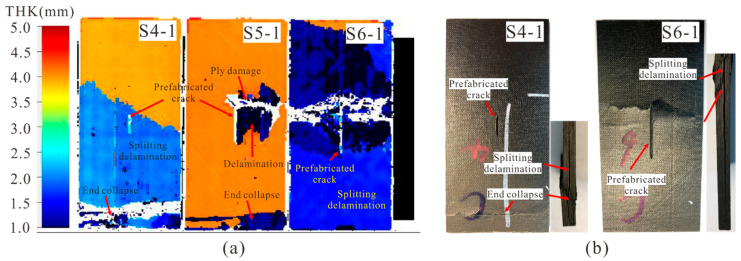
(**a**) C-scan results of specimens with 0° medium cracks after compression and (**b**) Failure modes comparison of S4-1 and S6-1.

Figure 8a shows the C-scan results of specimens with 0° medium cracks after the compression tests. The damage of S4-1 was similar to that of S3-1, which also presented as end crushing and a large area of split delamination. It should be noted that this large area of delamination did not occur at the depth of the prefabricated crack (the light blue region at a depth of 2 mm from the upper surface), and no visual damage was found near the crack, which indicates that the prefabricated crack did not propagate after the specimen failure. As the crack length increased to 18 mm (S5-1), slight material collapse was observed and the split area only appeared locally at the specimen end. Moreover, local delamination (the deep blue region at the depth between 1.0 mm and 1.5 mm) at the crack depth and ply damage (the white region) near the prefabricated crack tip area were found. This indicates that the delamination was caused by local ply damage or fracture. With the increase of the crack length to 26 mm, no crushing was detected at the end of S6-1. However, serious intra-laminar damage occurred at the crack tip, and a large area of delamination was induced at the crack depth (the deep blue region at the crack depth), which was obviously greater than S5-1. Meanwhile, the split delamination (the light blue region) was almost throughout the whole laminate as shown in Figure 8b. According to the acceptance criterion, the failure mode of S6-1 was the critical failure mode. When a more severe crack is prefabricated, the failure mode of the specimen will change from unaccepted to accepted.

**Figure 9:**
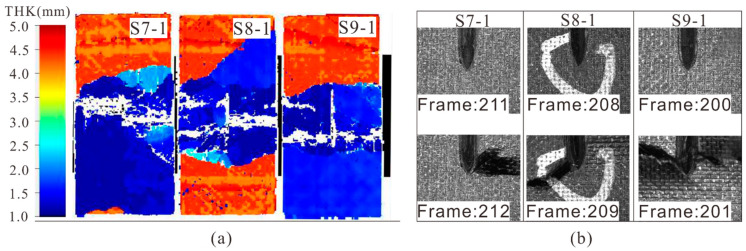
(**a**) C-scan results and (**b**) failure modes of specimens with 0° deep cracks after compression.

When the crack depth increased to 2.4 mm, a large area of delamination was found around the crack area and it was distributed in several locations along the laminate thickness (Figure 9a). The propagation of the crack tip was observed and obvious intra-laminar damage caused by crack tips was found (Figure 9b). The compression failure of three specimens occurred between the 201st frame and the 212th frame, and the corresponding strain was about 10,114–10,660 με. By comparing the failure strain with S3-1 (11,800 με), it could be seen that when the crack angle was 0°, the crack length and the crack depth had little effect on the compressive strengths of the laminates.

All the failure modes of specimens with 0° cracks are summarized in Table 2. When the crack length and depth were small, the failure mode was end crushing (unaccepted mode). With the increase of crack length and depth, the failure mode gradually changed to failure in the crack affected zone (accepted mode).

### 4.2. Crack Propagation of Specimens with 45° and 90° Cracks

**Figure 10:**
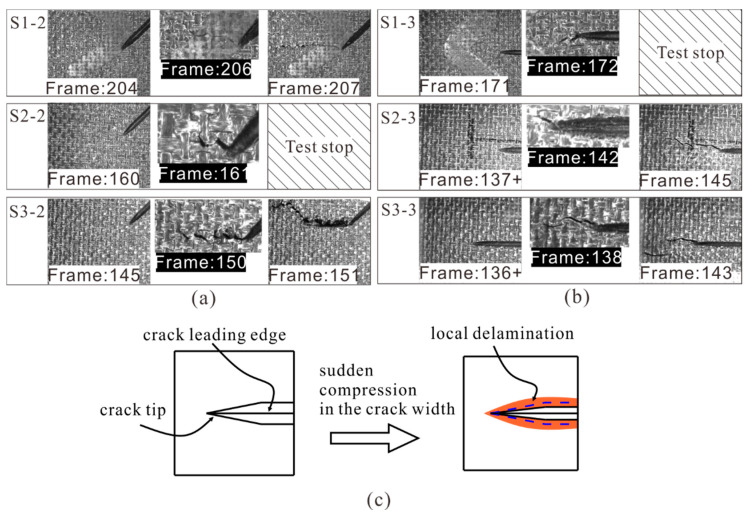
(**a**) 45° and (**b**) 90° shallow surface cracks propagation under compression; (**c**) illustration of the “sudden compression” phenomenon.

Except for specimens with 0° cracks, compression failure modes of the specimens were failure in the test area, which were accepted modes. Figure 10 shows the crack propagation of specimens with shallow surface cracks (*d* = 0.8 mm). In order to observe the crack propagation, S1-3 and S2-2 were unloaded once crack propagation was observed at the crack tip, and then C-scan testing was conducted to detect the internal damage. As shown in Figure 10a, the crack tips of specimens with 45° cracks (S1-2, S2-2, and S3-2) began to propagate transversely at the 204th, 160th, and 145th frame, respectively. For S1-2, the prefabricated crack started to grow at about 10,262 με, while for S3-2, the initial strain was about 7336 με, which decreased by about 28.5% due to the longer crack length. Then with the propagation of the crack, obvious material extrusion appeared at the crack tip, which indicates that inter-laminar delamination also occurred. Finally, the crack tip propagated rapidly to the sides of the specimen. Figure 10b shows the propagation of 90° cracks in specimens (S1-3, S2-3, and S3-3). The initial strains of crack propagation were lower than those of the 45° crack (S1-2, S2-2, and S3-2), which were about 6890–8626 με. In addition, the propagation of the 90° cracks was relatively slower compared with the 45° cracks. It is worth mentioning that in Figure 10b, the symbol “+” represents that a “sudden compression” phenomenon was observed in the width of the crack before crack tip propagation (137th to 138th frame for S2-3 and 136th to 137th frame for S3-3, about 6965 με). This phenomenon indicates that local delamination appeared first near the crack tip area, as shown in Figure 10c.

**Figure 11:**
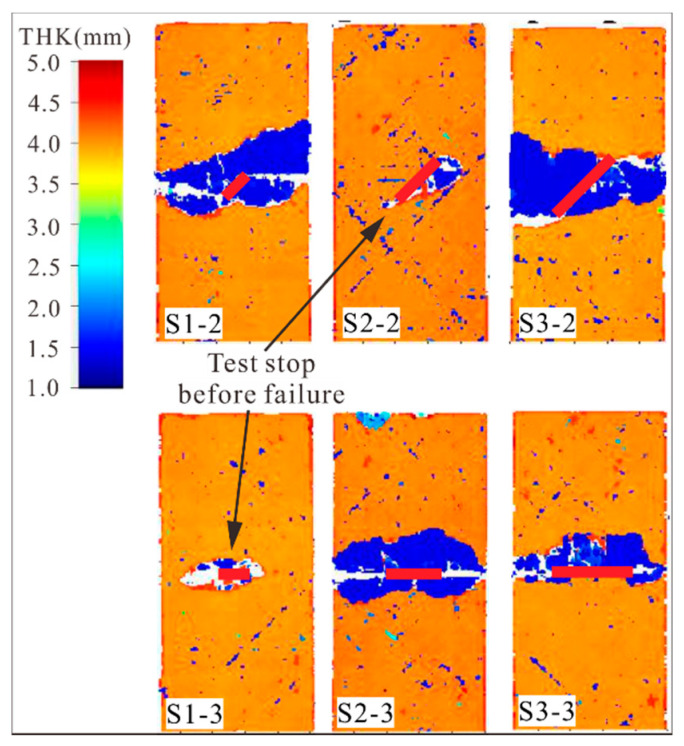
Delamination in specimens with shallow surface cracks after compression failure.

Figure 11 shows the C-scan results of specimens with shallow surface cracks after compression failure. For S1-3 and S2-2, surface ply fracture (the white region near crack tip) and local delamination (the blue region near crack tip) first occurred near the crack tips, indicating that they were caused by crack tip propagation. In contrast with the S1-3 and S2-2, there was a large area of delamination distributed in the prefabricated crack area. This proves that the delamination corresponding to compression failure of the specimen occurred after the propagation of the crack tip.

**Figure 12:**
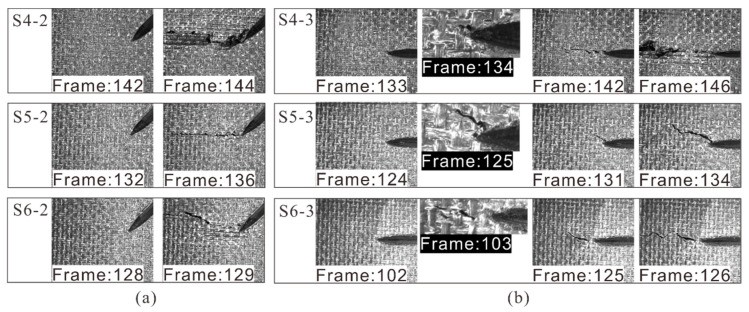
(**a**) 45° and (**b**) 90° medium cracks propagation under compression.

When the crack depth increased to 40% of the thickness, the initiation strain of 45° cracks was between 6493 με and 7188 με, which gradually decreased with the increase of crack length, as shown in Figure 12a. All the cracks propagated to the specimen sides in a few frames, which implies the instability of crack propagation. Obvious extrusion and dislocation of surface plies were observed during the crack propagation process. Figure 12b shows the crack growth in specimens with 90° cracks. The initial strain corresponding to the crack propagation decreased gradually with the increase of crack length, and the corresponding strain range was 5203–6741 με. Similar to shallow surface cracks, a stable crack growth process was found, the crack tip did not propagate with the load increase after initiation. After that, the crack rapidly propagated to the side of laminates, resulting in the failure of the specimen.

**Figure 13:**
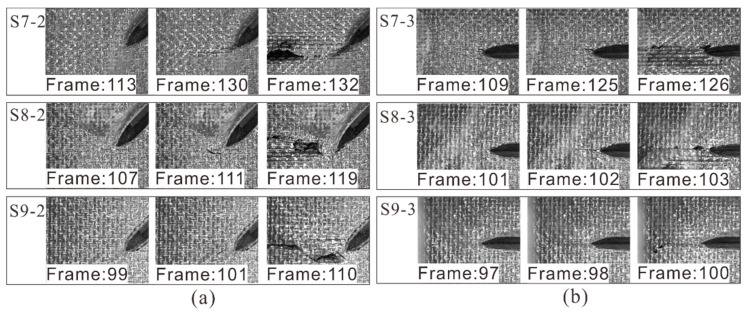
(**a**) 45° and (**b**) 90° deep cracks propagation under compression.

When the crack depth increased to 60% of the thickness, local deformation zones appeared in the 45° crack tip in compression (see Figure 13a), forming the local damage zone. These local zones were caused by large shear stress concentration at the crack tip due to the angle between the load and the crack being 45°. With the increase of the load, the zone expanded and triggered the crack tip propagation. It should be noted that these cracks grew more slowly than the previously mentioned 45° cracks, the reason being that the damage zone reduced the stress concentration at the crack tip [36]. It is more reasonable to consider the crack tip as local damage here due to the occurrence of local plasticity and complex failure mode. However, for 90° cracks (see Figure 13b), the crack tips propagated rapidly without obvious local deformation. The possible reason is that the deep crack made the in-plane stiffness of the laminate asymmetric, and an additional out-of-plane bending moment was induced during compression. This increased the longitudinal compression stress concentration at the crack tip, resulting in the rapid propagation of crack.

**Figure 14:**
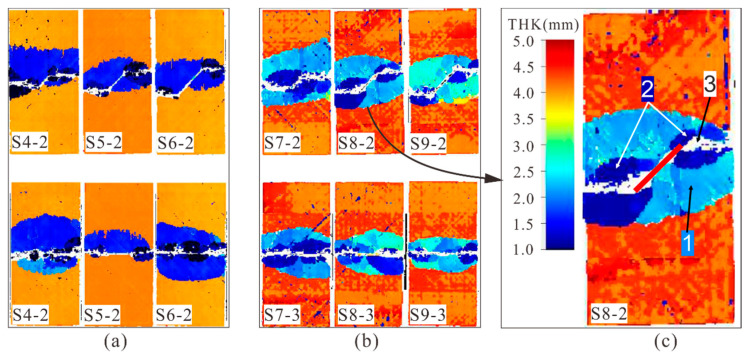
C-scan results of specimens after compression failure, (**a**) specimens with medium cracks, (**b**) deep cracks, and (**c**) typical results of S8-2.

Figure 14a,b shows the C-scan results of specimens with medium and deep cracks after failure. When the crack angle was the same, the projected delamination area decreased with the increase of crack lengths. Typical compression damage modes are shown in Figure 14c. It can be seen that along the thickness direction, delamination could be mainly divided into two types: (1) the large area of delamination surrounding the prefabricated crack, which propagated from the crack leading edge (the light blue region near 2.5 mm depth) or (2) the small area of delamination around the crack tips, which occurred with the crack tip growth (the dark blue region close to the upper surface). In addition, there were two transversely propagating damage bands from the crack tips to the sides of the laminate (the white region). Both the crack leading edge and the crack tip caused the delamination damage. The delamination caused by the crack leading edge was located at the depth of the crack and had a large area, whereas the delamination induced by the crack tip was limited in the region of crack tip propagation.

### 4.3. Typical Compression Failure Modes

**Figure 15:**
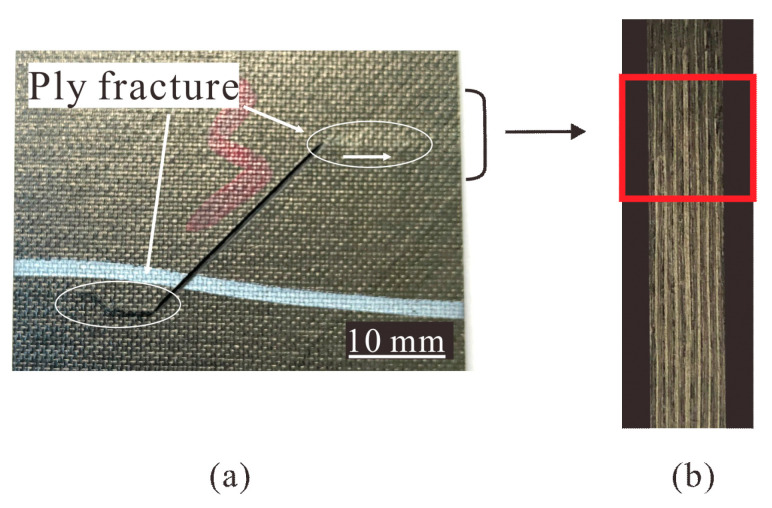
Compression failure modes of specimens (S3-2) with shallow surface cracks, (**a**) surface fracture, (**b**) failure mode (observed from the side).

Figure 15 shows the typical compression failure mode of specimens with shallow surface crack. Crack tips propagated first in the laminate, causing slight local ply fracture (Figure 15a). It can be seen in Figure 15b that after failure, damage on the side of the specimen was not obvious and the laminate remained almost intact.

**Figure 16:**
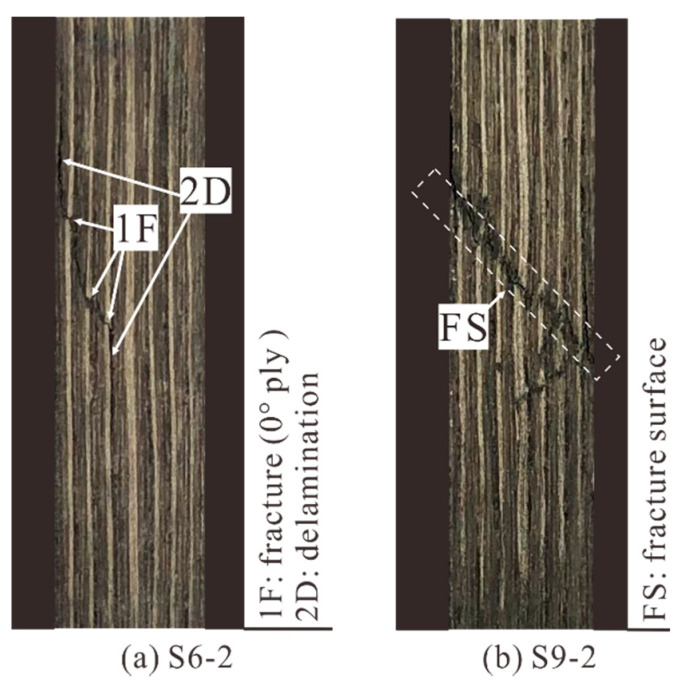
Compression failure modes of specimens (observed from the side), (**a**) with medium crack and (**b**) deep cracks.

Figure 16a shows the obvious ply fracture and delamination on the side of the specimen. The delamination mainly occurred at the same depth as the prefabricated crack, indicating that it was formed by the inter-laminar propagation of the crack leading edge. Moreover, the ply fracture was caused by the transverse propagation of crack tips. These two damage modes eventually formed a fractured sublaminate; however, the main laminate remained intact. With the crack depth further increasing, the failure process was basically the same as before, except that the main laminate broke. As shown in Figure 16b, the side of the specimen was completely broken, forming a 45° fracture surface.

## 5. Conclusions

In this paper, a new compression test fixture was designed to study the compression failure of composite laminates with prefabricated surface cracks, and compression tests were conducted to verify the validity of the test fixture and test method. Furthermore, the effects of three crack parameters on crack propagation behaviors and compression failure modes of laminates were compared. The conclusions are summarized as follows:

1. Two typical crack propagation modes were observed in composite laminates with surface cracks under compression, which were crack tip propagation (intra-laminar) and crack leading edge propagation (inter-laminar). The crack tip propagation led to local ply fracture and delamination, while the crack leading edge propagation caused delamination in a large area.
2. The initial strains of the crack tip propagation decreased with the increase of crack depths, lengths, and angles. When the crack angle was 0°, crack tip propagation did not easily occur and the compression failure load was high. When the crack angle was greater than 45°, the effect of the crack angle on crack propagation behaviors and failure modes was weakened.
3. The failure of laminates with prefabricated surface cracks under compression was that the crack tips propagated transversely first, and then a large area of delamination occurred from the crack leading edge. When the crack was shallow, the failure mode of the laminate was dominated by the large area of delamination. When the crack was medium or deep, the failure mode of the laminate was dominated by the propagation of crack tip and crack leading edge, which led to the fracture of the sublaminate or the whole laminate.

## Figures and Tables

**Table 1:** The test matrix for laminates with prefabricated surface cracks.

Specimen	*l* (mm)	*d* (mm)		*α* (°)
S1-1~3	10	0.8	(shallow)	0, 45, 90
S2-1~3	18	0.8		0, 45, 90
S3-1~3	26	0.8		0, 45, 90
S4-1~3	10	1.6	(medium)	0, 45, 90
S5-1~3	18	1.6		0, 45, 90
S6-1~3	26	1.6		0, 45, 90
S7-1~3	10	2.4	(deep)	0, 45, 90
S8-1~3	18	2.4		0, 45, 90
S9-1~3	26	2.4		0, 45, 90

**Table 2:** Failure modes of specimens with 0° cracks.

Specimen	Failure Mode	Note
S1-1	Not conducted	Unaccepted mode
S2-1	Not conducted	Unaccepted mode
S3-1	End crushing	Unaccepted mode
S4-1	End crushing	Unaccepted mode
S5-1	End crushing	Unaccepted mode
S6-1	Critical failure mode	Accepted mode
S7-1	Failure in the test area	Accepted mode
S8-1	Failure in the test area	Accepted mode
S9-1	Failure in the test area	Accepted mode

## Data Availability

The raw/processed data required to reproduce these findings cannot be shared at this time as the data also forms part of an ongoing study.

## Nomenclature

*E*_11_	Longitudinal elastic modulus
*E*_22_	Transverse elastic modulus
*ν*_12_	In-plane Poisson’s ratio
*G*_12_	In-plane shear modulus
*l*	Crack length
*d*	Crack depth
*α*	Angle between crack and loading direction
*F*_0_	Compression test preload
Δ*t*	Sampling interval
Δ*ε*	Strain increment
*ε*_0_	Initial strain corresponding to preload
*k*	Slope of load displacement curve
*A*	Extensional stiffness matrix
*N*	In-plane load vector
